# Altered MicroRNA Expression Profile in Exosomes during Osteogenic Differentiation of Human Bone Marrow-Derived Mesenchymal Stem Cells

**DOI:** 10.1371/journal.pone.0114627

**Published:** 2014-12-11

**Authors:** Ji-Feng Xu, Guang-hai Yang, Xiao-Hong Pan, Shui-Jun Zhang, Chen Zhao, Bin-Song Qiu, Hai-Feng Gu, Jian-Fei Hong, Li Cao, Yu Chen, Bing Xia, Qin Bi, Ya-Ping Wang

**Affiliations:** 1 Department of Orthopedics and Joint Surgery, Zhejiang Provincial People's Hospital, Hangzhou, 310014, PR China; 2 Department of Cardiology, Second Affiliated Hospital, College of Medicine, Zhejiang University, Hangzhou, 310009, PR China; 3 Department of Thoracic Surgery, Union Hospital, Tongji Medical College, Huazhong University of Science and Technology, Wuhan, 430022, PR China; Inserm U606 and University Paris Diderot, France

## Abstract

The physiological role of microRNAs (miRNAs) in osteoblast differentiation remains elusive. Exosomal miRNAs isolated from human bone marrow-derived mesenchymal stem cells (BMSCs) culture were profiled using miRNA arrays containing probes for 894 human matured miRNAs. Seventy-nine miRNAs (∼8.84%) could be detected in exosomes isolated from BMSC culture supernatants when normalized to endogenous control genes RNU44. Among them, nine exosomal miRNAs were up regulated and 4 miRNAs were under regulated significantly (Relative fold>2, p<0.05) when compared with the values at 0 day with maximum changes at 1 to 7 days. Five miRNAs (miR-199b, miR-218, miR-148a, miR-135b, and miR-221) were further validated and differentially expressed in the individual exosomal samples from hBMSCs cultured at different time points. Bioinformatic analysis by DIANA-mirPath demonstrated that RNA degradation, mRNA surveillance pathway, Wnt signaling pathway, RNA transport were the most prominent pathways enriched in quantiles with differential exosomal miRNA patterns related to osteogenic differentiation. These data demonstrated exosomal miRNA is a regulator of osteoblast differentiation.

## Introduction

The osteoblast, a cell type of a mesenchymal origin, plays a major role in skeletal development and bone formation [1], [2]. Understanding the regulatory mechanism of osteoblast differentiation is a prerequisite for developing strategies to treat bone loss diseases such as osteoporosis [3]–[5]. In the last two decades, progress in molecular and genetic research has uncovered various regulatory processes of osteoblast differentiation [1], [2], [4]. Central to this regulation are transcription factors; Runx2, Osterix, and β-catenin are, to date, the transcription factors known to be essential for osteoblast differentiation [2]. In addition, while some transcription factors, including C/EBP β, Smad1, and Smad5, bind to Runx2 and enhance its transcriptional activity, others, such as Twist, inhibit Runx2 transcriptional activity [6]. However, given the fact that the number of coding genes in vertebrates and invertebrates (which lack a skeleton) is comparable [7], there must be additional mechanisms for controlling skeletal development other than transcriptional regulation of gene expression.

Exosomes are present in most body fluids, and their composition differs depending on their cellular origin [8]–[10]. The presence of RNA has previously been confirmed in exosomes from saliva, plasma, and breast milk [11]. Exosomes can transfer genetic material to nearby cells, thereby affecting the function of the recipient cell [11]–[13]. However, their importance of exosomes in the regulation of osteoblast differentiation in vivo, if any, remains to be established. The presence of microRNA (miRNA) in exosomes from certain bodily fluids, including saliva, has also been confirmed [13], [14]. MiRNAs are small (22-nt) endogenous noncoding RNAs that anneal to the 3′UTR of target mRNAs to mediate inhibition of translation and lower protein levels [15]. In addition, miRNAs have emerged as key negative regulators of diverse biological and pathological processes, including developmental timing, organogenesis, apoptosis, cell proliferation, and differentiation [16] and in the control of tumorigenesis [17], [18]. It remains to be established how specific miRNAs contribute to regulate the onset of a tissue-specific phenotype in response to a multifunctional morphogen. Previous reports have implicated the potential roles of miRNAs in the differentiation of osteoclasts and osteoblasts [19]–[21]. However, alterations of exosomal miRNA content in osteoblast differentiation have not yet been described. The primary goal of this study was to characterize differences in exosomal miRNA during osteogenic differentiation of human BMSCs, and to explore their biological functions.

## Materials and Methods

### Isolation and culture of human BMSCs

Human BMSCs were isolated and expanded using a modification of methods previously reported [22]. The study has been approved by the Ethical Committee of Zhejiang Provincial People's Hospital; written informed consent was obtained from all subjects or their parents in the case of children. This work received approval from the institution ethics committee and conformed to the tenets of the Declaration of Helsinki. Eleven subjects (F/M = 6/5; Age = 25±7) are with no metabolic disease, inherited diseases and other diseases which may affect the current study. Bone marrow aspirates were obtained during routine orthopedic surgical procedures. Marrow aspirates (20 ml volumes) were harvested using a bone marrow biopsy needle inserted through the cortical bone; aspirates were immediately resuspended in α-MEM (Life technologies; Carlsbad, CA) containing 10% fetal bovine serum (FBS), 100 U/ml penicillin and 100 mg/l streptomycin (Life technologies), and cultured in a humidified 37°C/5% CO_2_ incubator. hBMSC were selected on the basis of adhesion and proliferation on tissue culture plastic substrate. After 3 days, nonadherent cells were removed by 2–3 washes with PBS and adherent cells further cultured in α-MEM until 90% confluence. The obtained BMSCs were cultured and expanded for further experiments. The BMSCs prior to passage four were used in the following experiments. To induce osteoblastic differentiation, BMSCs were cultured in an osteogenic medium (α-MEM supplemented with 10% FBS, 50 mg/ml L-ascorbic acid, 10 mM glycerophosphate and 100 nM dexamethasone and antibiotics (Sigma; St. Louis, MO)) for 7 days.

### Isolation of exosomes

Exosomes were isolated as described before [23]. In brief, BMSCs culture supernatants were subjected to successive centrifugations of 3,042× g (20 min) and 10,000× g (30 min). Exosomes were then pelleted at 64,000× g for 110 min, using an SW28 rotor (Beckman Coulter Instruments). Exosome pellets were resuspended in 0.32 M sucrose and centrifuged at 100,000× g for 1 h (SW60Ti rotor; Beckman Coulter Instruments). The exosome pellet was then resuspended in PBS.

### Exosome characterization by FACS analyses

hBMSC culture supernatants were added directly to Dynabeads (2 mL/mL beads) coated with anti-MHC class II (MHCII) antibodies (clone HKB1; Invitrogen/Dynal, Paisley, United Kingdom), as previously described [24]. Beads were labeled with fluorescein isothiocyanate-labeled anti-MHC class I, anti-MHC class II, CD63, CD86 and phycoerythrin-labeled anti-CD54 or isotype-matched controls (BioLegend, San Diego, Calif). Samples were analyzed in a FACS Calibur (BD Biosciences, San Jose, CA) by using forward scatter/side scatter bead gating, and mean fluorescence intensity (MFI) ratios were calculated as the geometric mean of the marker divided by the geometric mean of the isotype control.

### RNA processing

RNA was extracted and separated into small RNA (including miRNAs, 18–200 nt) and large RNA (>200 nt) fractions by using Nucleospin miRNA, according to the manufacturer's instructions. RNA quality was assessed by using UV 260/280 and 230/260 absorbance ratios obtained by using Nanodrop (Thermo Scientific, Wilmington, NC), resulting in a mean 260/280 ratio of 1.95. RNA size distribution was examined on RNA Pico LabChips (Agilent Technologies, Palo Alto, CA) processed on the Agilent 2100 Bioanalyzer small RNA electrophoresis program.

### Global miRNA expression profiling

An aliquot of 1 µL was used for validation by means of quantitative RT-PCR, and the rest was concentrated (SpeedVac, Thermo Fisher) to a volume of 4 µL and used for amplification. RNA was labeled using the miRCURY LNA microRNA power labeling kit (Exiqon, Woburn, Mass), according to the manufacturer's protocol. Labeled RNA was hybridized to affymetrix platform for miRNA expression analysis (Genechip miRNA 3.0 array) was used to obtain miRNA profiles. Microarray data are available in the ArrayExpress with the identification of ArrayExpress accession: E-MTAB-2977.

### MicroRNA target prediction and pathway analysis

DIANA-mirPath [25], a web-based application, was introduced to perform the enrichment analysis of predicted target genes by one or more miRNAs in biological pathways. Two algorithms were used to predict miRNA targets, namely, microT-CDS [26], [27] and miRTarBase [28]. The software performs an enrichment analysis of multiple miRNA target genes to all known KEGG pathways. The combinatorial effect of co-expressed miRNAs in the modulation of a given pathway is taken into account by the simultaneous analysis of multiple miRNAs. The graphical output of the program provides an overview of the parts of the pathway modulated by selected miRNAs, facilitating the interpretation and presentation of the analysis results. The statistical significance value associated with the identified biological pathways was calculated by the mirPath. The software is available at http://microrna.gr/mirpath.

### TaqMan miRNA Assay for Individual miRNAs

Independent sets of exosome samples from hBMSC cultured and stimulated by different time points were used for qPCR confirmation. Total RNA was isolated as described earlier. nine up regulated miRNAs (let-7a, miR-199b, miR-218, miR-148a, miR-135b, miR-203, miR-219, miR-299-5p, and miR-302b) and five down regulated miRNAs (miR-221, miR-155, miR-885-5p, miR-181a, and miR-320c) from miRNA array were further quantitated by TaqMan miRNA assays (Applied Biosystems).

### Quantitative real-time PCR (QPCR)

Candidate mRNA transcripts levels were measured by real-time PCR using gene-specific primers; For real-time PCR, the diluted cDNA was amplified for 40 cycles with a master mix (SYBR Green Supermix; Applied Biosystem) using a thermo-cycler (Bio-Rad, Hercules, CA, USA). Melting curve analysis was done at the end of the reaction to assess the quality of the final PCR products. The threshold cycle C(t) values were calculated by fixing the basal fluorescence at 0.05 units. Three replicates were used for each sample and the average C(t) value was calculated. The ΔC(t) values were calculated as C(t) sample – C(t) controls. The N-fold increase or decrease in expression was calculated by the ΔΔCt method using the C(t) controls value as the reference point.

### Statistical analysis

The microarray dataset was normalized by using quantile normalization [29]. No background subtraction was performed, and the median feature pixel intensity was used as the raw signal before normalization. miRNAs with a log2 expression value of less than 5.5, corresponding to a signal-to-noise ratio of less than 2, were excluded from all statistical analyses. Univariate statistical analyses were performed with a 2-way ANOVA linear model, and P value correction to account for a high false-positive rate was performed by using the false discovery rate method according to Hochberg and Benjamini [30] with the R package limma in Bioconductor [31], [32]. Heatmap of differentially expressed miRNAs and between-group statistic analysis were performed by R software. The Mann-Whitney test was performed to determine the significance of miRNA levels for independent validation. All data were expressed as mean ± SEM, unless otherwise specified. P values less than 0.05 were considered statistically significant.

## Results

### FACS characterization of exosomes

In the process of human BMSCs osteogenic differentiation, the mRNA and protein expression levels of osteoblastic target genes, such as alkaline phosphatase (ALP), bone morphogenetic protein 2 (BMP-2), and platelet-derived factor alpha polypeptide (PDGF-α) at most time points increased significantly when compared with the values at 0 hour with maximum changes at 1 to 7 days (S1 Figure). To confirm that the structures studied indeed are exosomes, they were examined by flow cytometric analysis. Human BSMC culture supernatants were treated with Dynabeads to detect surface proteins. Pilot experiments showed the identity of the studied vesicles was confirmed as exosomes by FACS analysis by specifically binding to latex beads coated with anti-CD63 (S2 Figure), which demonstrated the presence of the surface protein CD63 - a commonly used marker of exosomes. Further analysis indicated that exosomes from all samples showed the presence of MHCII. However, MHC class I, CD54, and CD86 were not detected (S2 Figure). No significant differences were seen between groups.

### MiRNAs were differentially expressed in exosomes during osteogenic differentiation of human BMSCs

**Figure 1:**
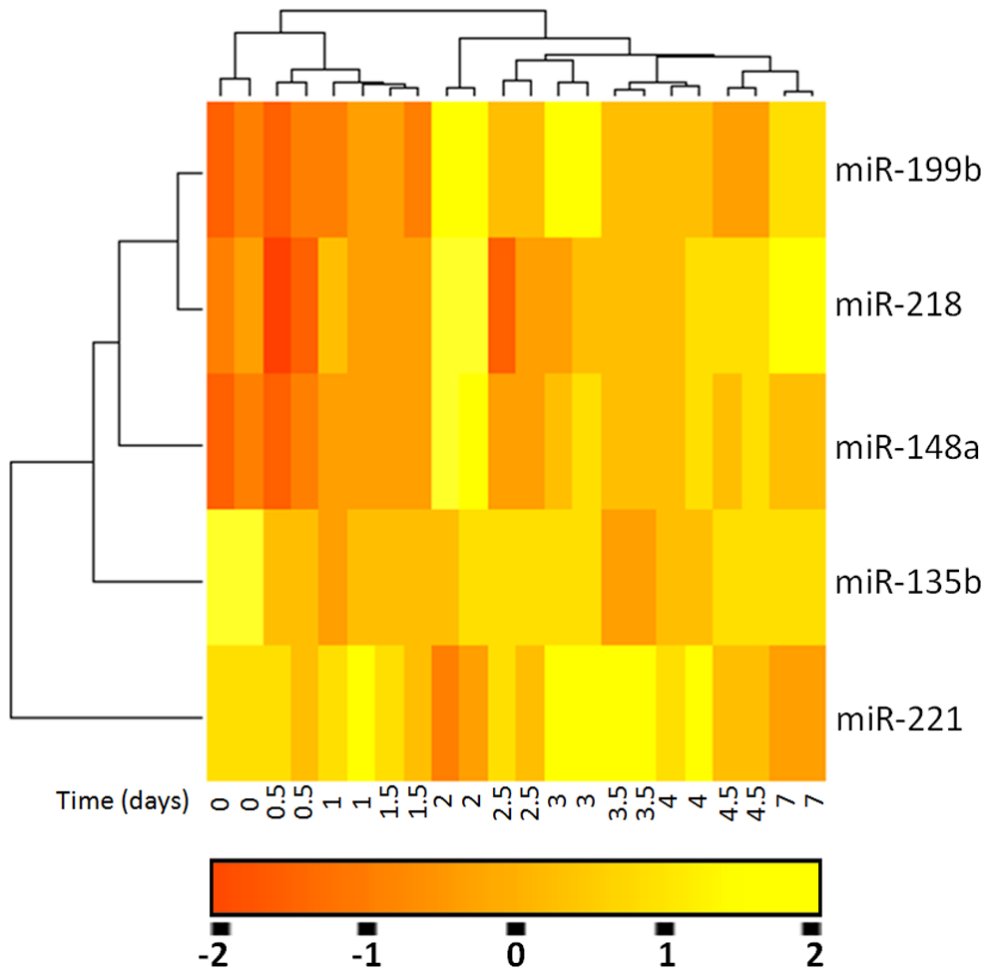
miRNAs were differentially expressed in exosomes during osteogenic differentiation of human BMSCs. Heatmap representation of the mean fold change in osteogenic differentiation related differential miRNA signature. Two-dimensional grid matrix displaying 5 differential miRNAs (miR-199b, miR-218, miR-148a, miR-135b, and miR-221) was obtained by the functional heat-map in R. Columns refer to time course comparison: human BMSC culture at 0, 0.5, 1, 1.5, 2, 2.5, 3, 3.5, 4, 4.5, 7 days. Rows stand for the 5 differential miRNAs. Each entry of the grid refers to relative fold (log2) between the expression level of a given miRNA in exosome relative to RNU44 in human BMSCs. The color of each entry is determined by the value of that fold difference, ranging from red (negative values) to yellow (positive values).

In an initial effort to identify differentially expressed miRNA in exosomes during osteogenic differentiation, we profiled the expression of 894 miRNA by using Agilent custom miRNA v3.5 arrays. The high sensitivity and specificity of this method have been well established [33]. The miRNA raw data was firstly normalized by using quantile normalization. No background subtraction was performed, and the median feature pixel intensity was used as the raw signal before normalization. miRNAs with a log2 expression value of less than 5.5, corresponding to a signal-to-noise ratio of less than 2, were excluded from all statistical analyses. To investigate the relative abundances of the exosomal miRNAs detected, they were then normalized in each sample to RNU44 in BMSCs. The data indicated that 79 miRNAs (∼8.84%) could be detected (**S1 Table**). The study revealed differential expression of 14 exosomal miRNAs in BMSC culture supernatant when cultured at 0, 0.5, 1, 1.5, 2, 2.5, 3, 3.5, 4, 4.5, 7 days. Among them, we found that 5 miRNAs (miR-199b, miR-218, miR-148a, miR-135b, and miR-221) were more than 2-fold change in exosomes isolated from BMSCs culture when compared with the maximum changes at 0.5 to 7 days with the values at 0 day. (**Table 1**). In more detail, the expression level of miR-199b in BSMC exosomes was 3.75±0.81 folds increase at day 4 of osteogenic differentiation compared to that of day 0. miR-218 has a 2.81±1.01 over expression on day 3 osteogenic differentiation relative to that of day 0. There was a 3.11±0.94 increase of expression levels of miR-148a on day 1 compared to that of day 0. miR-135b has 2.99±o.77 up regulation on day 2.5 compared to that of day 0. In addition, a significantly decrease (0.31±0.14) in miR-221 expression level on day 7 compared to that of day 0. The heatmap, which represented the mean fold change of differential miRNA signatures, was generated by these 5 differentially expressed miRNAs (**Fig. 1**). These data indicated that exosomal miRNAs were differentially expressed in human BMSCs when cultured at 0, 0.5, 1, 1.5, 2, 2.5, 3, 3.5, 4, 4.5, 7 days. Further, these data indicated that our preliminary screening assay on aberrantly expressed miRNAs for osteogenic differentiation in exosomes was reliable and feasible method.

**Table 1:** Differential miRNA expression in exosomes of human BMSCs during osteogenic differentiation.

Maximum changes at 0.5 to 7 days vs. 0 day	microRNA	Fold change	Adjusted P
Up-regulated	let-7a	2.239	0.0145
	miR-199b	2.928	0.0420
	miR-218	2.288	0.0068
	miR-148a	2.284	0.0471
	miR-135b	2.558	0.0254
	miR-203	7.275	0.0448
	miR-219	2.164	0.0419
	miR-299-5p	2.763	0.0294
	miR-302b	2.075	0.0216
Down-regulated	miR-221	0.172	0.0005
	miR-155	0.132	0.0466
	miR-885-5p	0.375	0.0134
	miR-181a	0.155	0.0254
	miR-320c	0.264	0.0101

### Validation of miRNA array expression using independent samples

**Figure 2:**
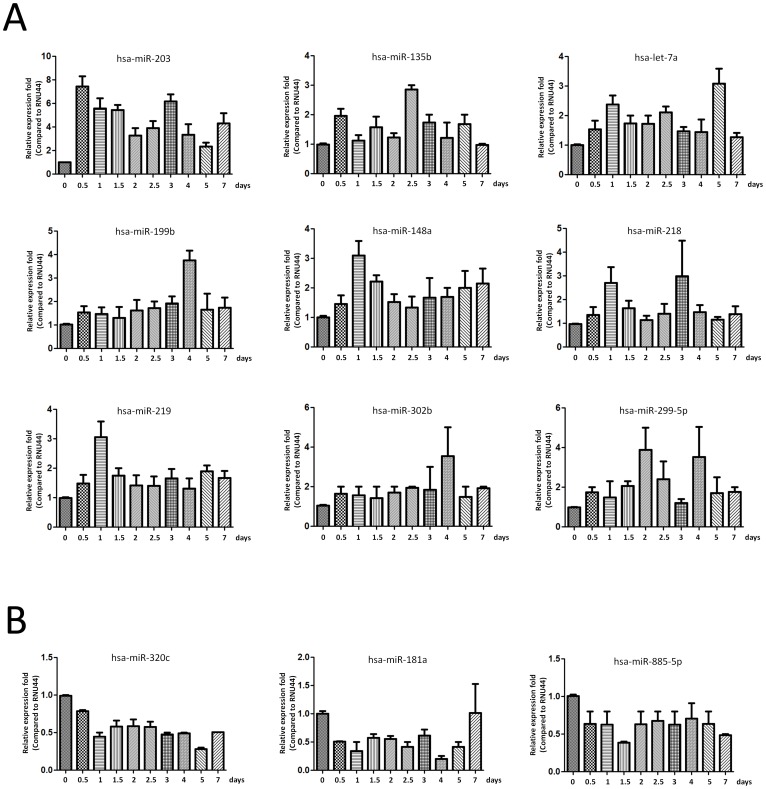
Validation of miRNA array expression using independent samples. TaqMan real-time RT-PCR to validate the expression levels of nine up regulated miRNAs, including let-7a, miR-199b, miR-218, miR-148a, miR-135b, miR-203, miR-219, miR-299-5p, and miR-302b (A) and three down regulated miRNAs, including miR-885-5p, miR-181a, and miR-320c (B) from miRNA array were selected for further validation using individual exosomal samples from BMSCs when cultured at 0, 0.5, 1, 1.5, 2, 2.5, 3, 4, 5, 7 days. Data shown are representative of three independent experiments and are shown as mean ± SEM.

We employed TaqMan real-time RT-PCR to validate the expression levels of the dysregulated miRNAs from microRNA assay. Nine up regulated miRNAs (let-7a, miR-199b, miR-218, miR-148a, miR-135b, miR-203, miR-219, miR-299-5p, and miR-302b) and five down regulated miRNAs (miR-221, miR-155, miR-885-5p, miR-181a, and miR-320c) from miRNA array were selected for further validation using individual exosomal samples from BMSCs when cultured at 0, 0.5, 1, 1.5, 2, 2.5, 3, 3.5, 4, 4.5, 7 days. In agreement with the preliminary data from microRNA assay, let-7a, miR-199b, miR-218, miR-148a, miR-135b, miR-203, miR-219, miR-299-5p, and miR-302b were significantly increased in individual exosomal samples from BMSCs when compared with the changes at 0.5 to 7 days with the values at 0 hour. While miR-221, miR-155, miR-885-5p, miR-181a, and miR-320c were significantly under expressed in individual exosomal samples along with the time course at 0, 0.5, 1, 1.5, 2, 2.5, 3, 3.5, 4, 4.5, 7 days (**Fig. 2**). Taken together, independent TaqMan real-time qPCR expression results confirmed the validity of differentially expressed exosomal miRNAs identified by human miRNA assay and our results defined a profile of dysregulated exosomal miRNA signatures related to osteogenic differentiation, which revealed that these miRNAs may have a functional role in the pathogenesis of osteogenic dysfunction.

### Comparative pathway analyses

**Figure 3:**
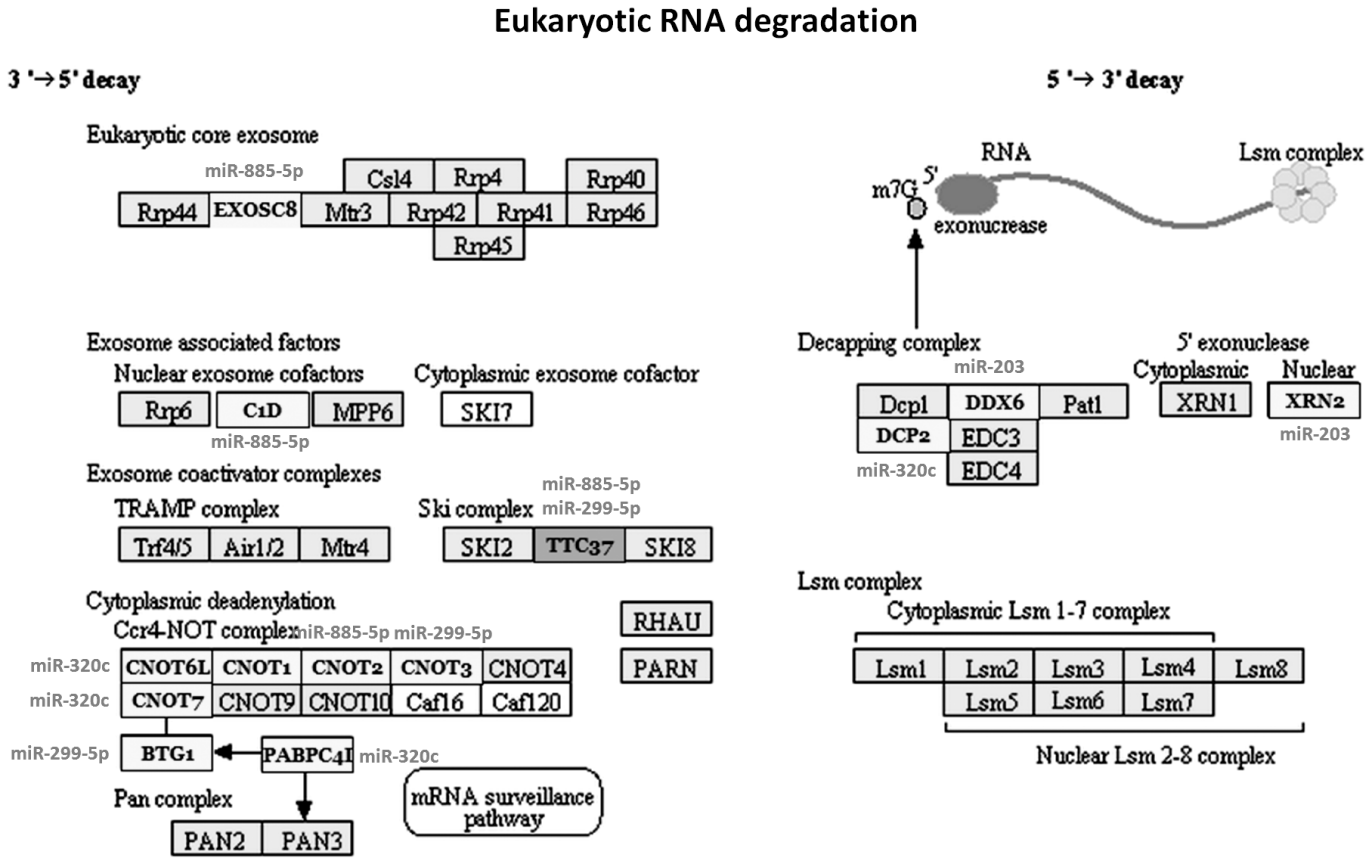
Diagram of RNA degradation. Four differentially expressed miRNAs (including miR-203, miR-299-5p, miR-885-5p, and miR-320c) were selected as examples for examining miRNA-mRNA relationships in RNA degradation.

In order to examine which biologic pathways were affected during osteogenic differentiation of hBMSCs, we applied DIANA-mirPath on osteogenic differentiation related dysregulated exosmal miRNA signatures for further investigation. Twenty-three KEGG biological processes were significantly enriched (p<0.05, FDR corrected) among differentially expressed exosomal miRNAs in hBMSCs when compared with the changes at 0.5 to 7 days with the values at 0 hour. Among them, Biotin metabolism (p = 1.301E-08), RNA degradation (p = 1.569E-07), mRNA surveillance pathway (p = 1.637E-07), Wnt signaling pathway (p = 9.512E-05), Tight junction (p = 1.261E-04), B cell receptor signaling pathway (p = 2.776E-03), Adipocytokine signaling pathway (p = 3.661E-03), Adherens junction (p = 4.080E-03), Leukocyte transendothelial migration (p = 1.911E-02), RNA transport (p = 3.029E-02) were the most prominent pathways enriched in quantiles with related differentially expressed miRNA patterns related to osteogenic differentiation (**Table 2** **,** **Figs. 3** **–** **5**), suggesting that these biologic pathways were involved in osteogenic differentiation of hBMSC and bone development.

**Figure 4:**
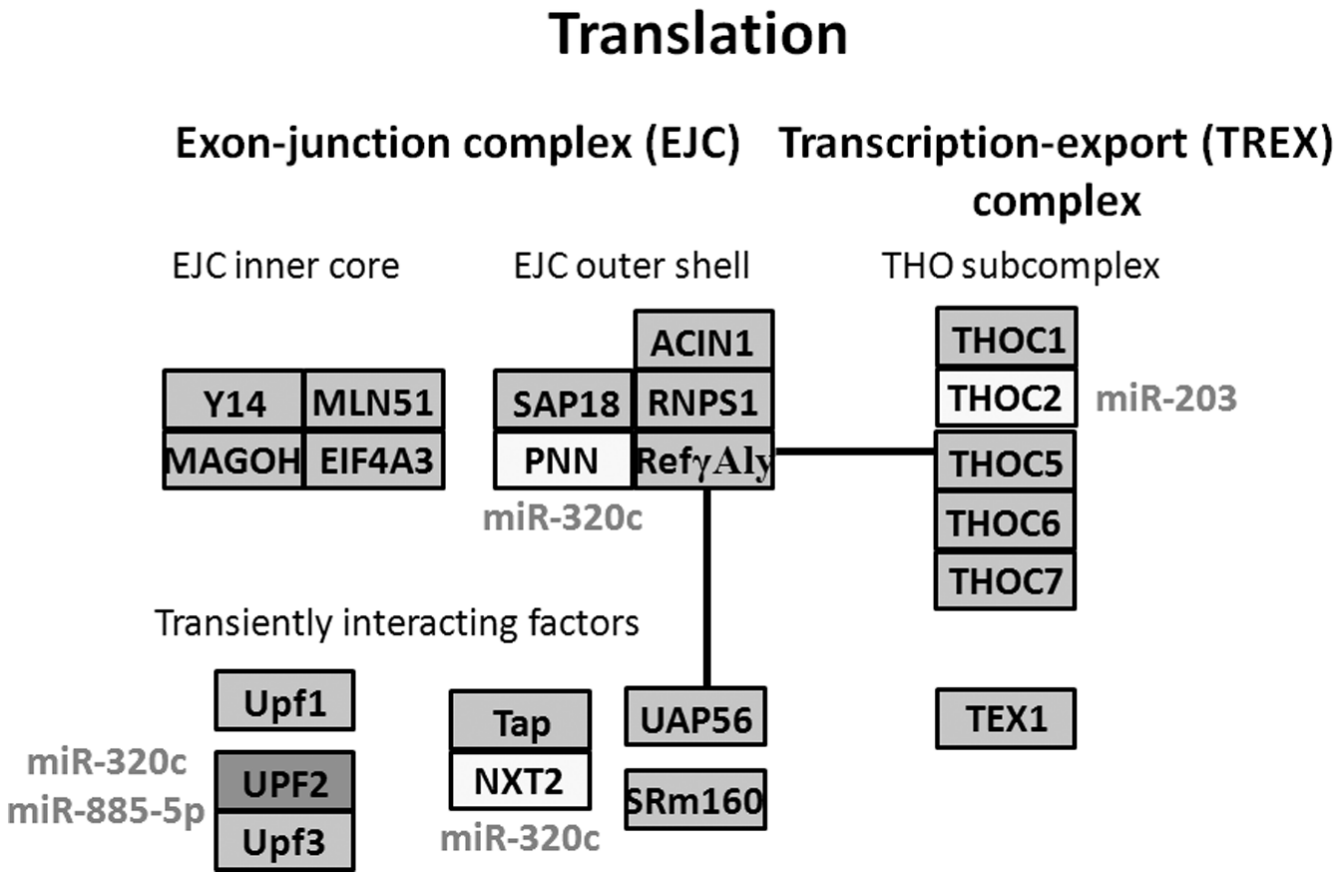
Diagram of RNA transport. Three under-expressed miRNAs (miR-203, miR-885-5p, and miR-320c) were predicted to participate in RNA transport pathway through regulating their potential gene targets.

**Figure 5:**
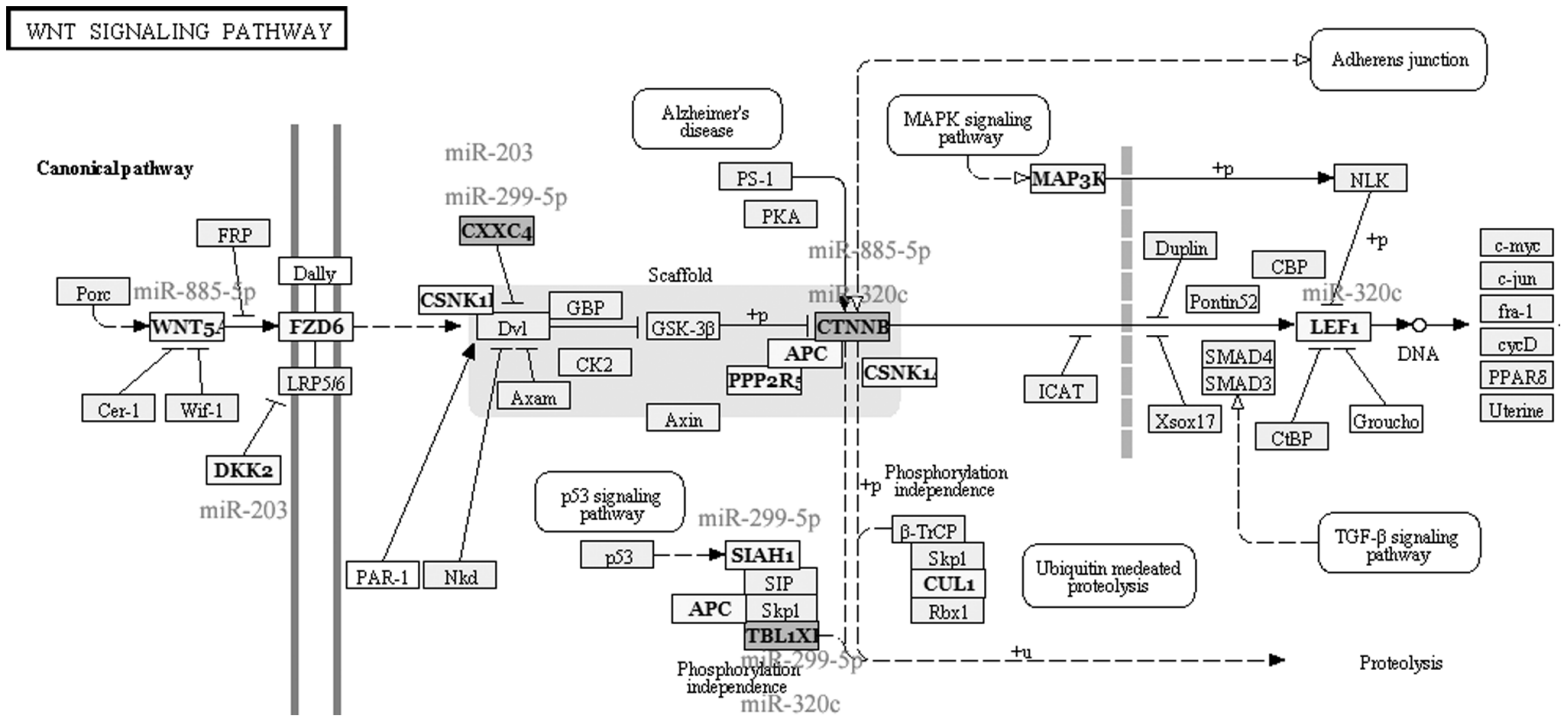
Diagram of Wnt signaling pathway. Four differentially expressed miRNAs (including miR-203, miR-299-5p, miR-885-5p, and miR-320c) were predicted to participate in Wnt signaling pathway through regulating their potential gene targets.

**Table 2:** Biologic pathways enriched by differentially expressed exosomal miRNAs.

KEGG pathway	P-value, FDR corrected	#genes	#miRNAs
Biotin metabolism (hsa00780)	1.301E-08	2	2
RNA degradation (hsa03018)	1.569E-07	16	4
mRNA surveillance pathway (hsa03015)	1.637E-07	19	4
Ubiquitin mediated proteolysis (hsa04120)	1.799E-05	22	4
mTOR signaling pathway (hsa04150)	2.600E-05	13	4
PI3K-Akt signaling pathway (hsa04151)	3.421E-05	42	4
Insulin signaling pathway (hsa04910)	5.328E-05	21	4
Aldosterone-regulated sodium reabsorption (hsa04960)	6.102E-05	9	3
Wnt signaling pathway (hsa04310)	9.512E-05	23	4
MAPK signaling pathway (hsa04010)	1.261E-04	33	4
Tight junction (hsa04530)	1.261E-04	20	4
p53 signaling pathway (hsa04115)	1.544E-04	12	3
Focal adhesion (hsa04510)	5.317E-04	26	4
ErbB signaling pathway (hsa04012)	1.182E-03	15	4
B cell receptor signaling pathway (hsa04662)	2.776E-03	12	3
Adipocytokine signaling pathway (hsa04920)	3.661E-03	10	3
Adherens junction (hsa04520)	4.080E-03	12	4
Pantothenate and CoA biosynthesis (hsa00770)	1.598E-02	4	2
Leukocyte transendothelial migration (hsa04670)	1.911E-02	16	4
Valine, leucine and isoleucine biosynthesis (hsa00290)	3.029E-02	1	1
RNA transport (hsa03013)	3.029E-02	18	4
Gap junction (hsa04540)	3.550E-02	12	4
VEGF signaling pathway (hsa04370)	3.813E-02	9	3

### mRNA transcripts in exosomes during osteogenic differentiation of human BMSCs

**Figure 6:**
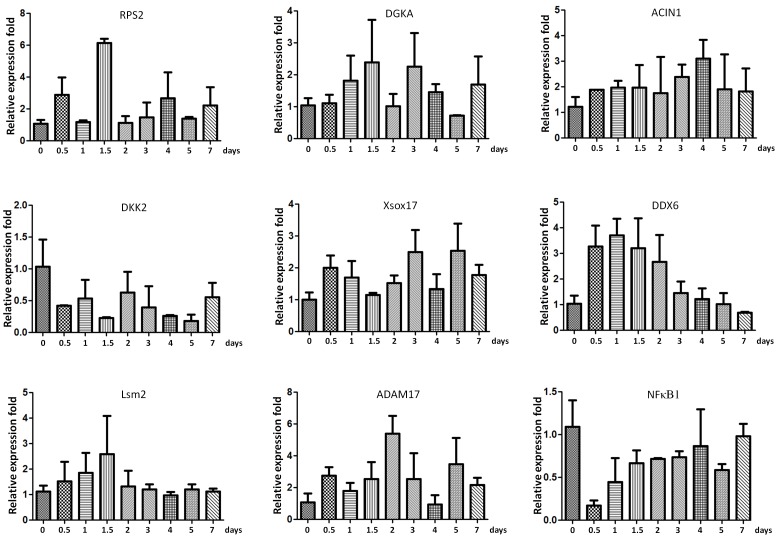
mRNA transcripts in exosomes during osteogenic differentiation of human BMSCs. Seven mRNAs (RPS2, DGKA, ACIN1, DKK2, Xsox17, DDX6, and Lsm2) were selected for qRT-PCR experiments using BSMC exosomal RNA from twelve independent subjects. In addition, qRT-PCR to detect the expression levels of ADAM17 and NFκB1 in exosomes from the osteogenic differentiated BSMCs. Data shown are representative of three independent experiments and are shown as mean ± SEM.

In order to analyze the other components in the exosomes isolated from osteogenic differentiated BSMC, we tried to examine the presence of messenger RNAs (mRNAs) associated with exosomes and elucidate their roles during BSMC osteogenic differentiation and cell-cell communication. The BSMC exosomal RNA was heterogeneous in size, but contained no or little ribosomal RNA (18S- and 28S- rRNA) compared to the donor cells. We then employed qRT-PCR to detect the expression of mRNA transcripts isolated from exosomes. Twenty-four potential genes in the discovered biologic pathways enriched by differentially expressed exosomal miRNAs, including RNA degradation, mRNA surveillance pathway, Wnt signaling pathway, Tight junction, Adipocytokine signaling pathway, Adherens junction, Leukocyte transendothelial migration, and RNA transport, were selected for qRT-PCR experiments using BSMC exosomal RNA from twelve independent subjects. Seven mRNAs (RPS2, DGKA, ACIN1, DKK2, Xsox17, DDX6, and Lsm2) were found to be expressed and significantly differentially expressed over time in differentiated BSMC exosomes (**Fig. 6**). In addition, we also observed that expression levels of two NFκB related genes, including ADAM17 and NFκB1, were detected and dysregulated expressed in exosomes from the osteogenic differentiated BSMCs using qRT-PCR assay (**Fig. 6**). Taken together, mRNA transcripts in exosomes might be involved in BSMC osteogenic differentiation and cell-cell communication.

### miR-885-5p regulates BMP2-induced osteogenic differentiation

**Figure 7:**
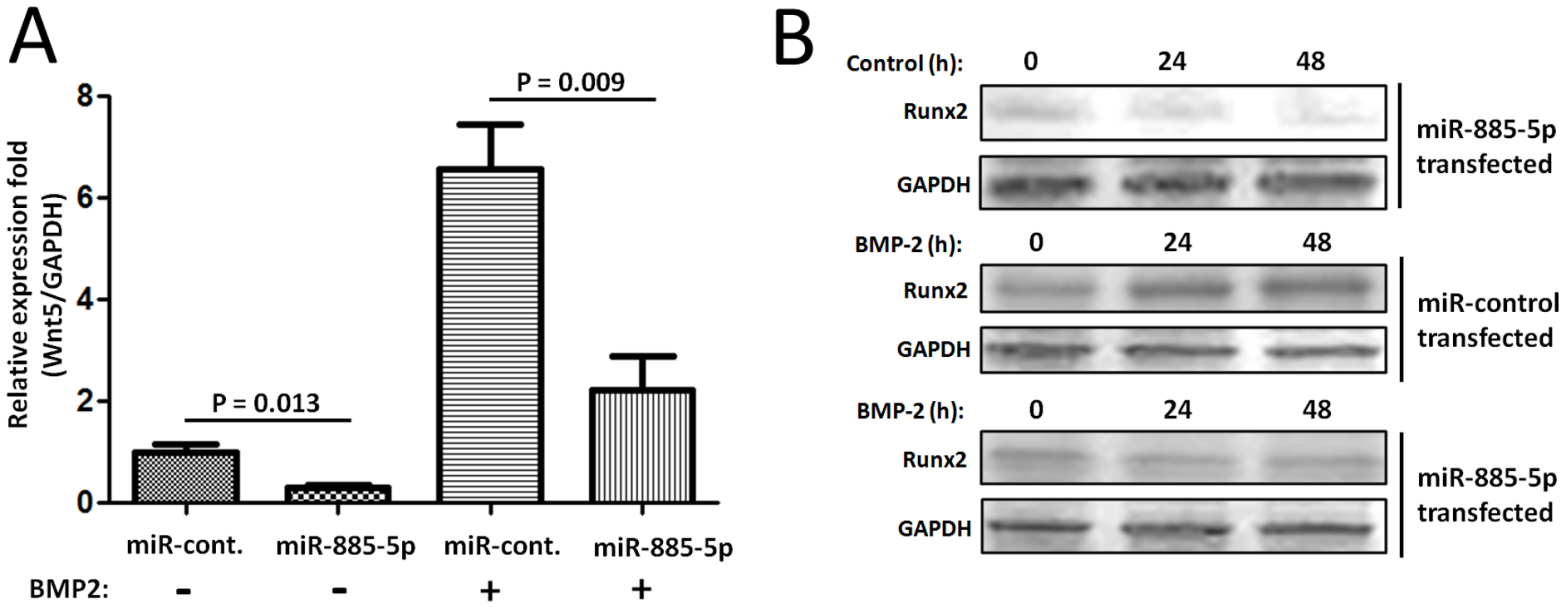
miR-885-5p regulates BMP2-induced osteogenic differentiation. (A). RT-qPCR to detect the expression of Wnt5 mRNA level when miR-885-5p over expression. Data shown are representative of three independent experiments and are shown as mean ± SEM. (B). Western Blot to detect Runx2 protein level in BSMCs were treated with BMP2 for 24 and 48 h after expressing miR-885-5p and miR-Control for 24, 48 h. Data shown are representative of three independent experiments.

We further tried to uncover the potential functions of exosomal miRNAs in BSMCs differentiation. Wnt5a, a classical noncanonical Wnt, was recently reported as a critical component of BMP2-mediated osteogenic differentiation [34]. Further RT-qPCR confirmed that miR-885-5p over expression leads to down-regulated expression of Wnt5 mRNA level (**Fig. 7A**). To determine whether exosomal miR-885-5p are linked to BMP2-induced BSMCs osteogenic differentiation through their targets, BSMCs were treated with BMP2 for 24 and 48 h after expressing miR-885-5p for 12 h. As expected, Runx2 protein level was enhanced by BMP2 in cells transfected with miR-Control (**Fig. 7B**). However, miR-885-5p expression restrained the increase of Runx2 protein after BMP2 treatment (**Fig. 7B**). Our results suggest that miR-885-5p functions as a negative regulator of osteogenic differentiation of BSMCs by repressing Runx2 and thereby inhibiting expression of osteoblast-related genes.

## Discussion

Bone organogenesis is a complex process involving the differentiation and crosstalk of multiple cell types for formation and remodeling of the skeleton [35]. MicroRNAs are critical post-transcriptional regulators of gene expression that control osteoblast mediated bone formation and osteoclast-related bone remodeling [19], [36]. Deregulation of miRNA mediated mechanisms is emerging as an important pathological factor in bone degeneration (eg, osteoporosis) and other bone-related diseases. Increasing evidence supports the importance of miRNA regulation in osteogenic differentiation of BMSCs, but the regulatory mechanism has so far been poorly defined. In this study we have, for the first time, verified the presence of miRNA in exosomes isolated from the supernatant of human BMSCs culture. Furthermore, we revealed alterations in the exosomal miRNA profiles from osteogenic differentiated BMSCs at different time points, suggesting an intrinsic dysregulation of exosome miRNA content during osteogenic differentiation.

Exosomes contain various molecular constituents of their cell of origin, including proteins and RNA. Although the exosomal protein composition varies with the cell and tissue of origin, most exosomes contain an evolutionary-conserved common set of protein molecules. The RNA molecules in exosomes include mRNA and miRNA [37]. In this study we have, for the first time, verified the presence of miRNA in exosomes during BMSCs osteogenic differentiation. Furthermore, we found that let-7a, miR-199b, miR-218, miR-148a, miR-135b, miR-203, miR-219, miR-299-5p, and miR-302b were significantly increased in individual exosomal samples from human BMSCs. While miR-221, miR-155, miR-885-5p, miR-181a, and miR-320c were significantly under expressed in individual exosomal samples. Furthermore, we examined the presence of messenger RNAs associated with exosomes and elucidate their roles during BSMC osteogenic differentiation. Seven mRNAs (RPS2, DGKA, ACIN1, DKK2, Xsox17, DDX6, and Lsm2) were found to be expressed and significantly differentially expressed over time in differentiated BSMC exosomes. There were numerous evidences for these dysregulated exosomes-associated miRNAs, which have proven to play important roles during osteogenic differentiation of BSMCs. MicroRNA, let-7, was reported to enhance osteogenesis and bone formation while repressing adipogenesis of human stromal/mesenchymal stem cells by regulating HMGA2 [38]. MSCs with a high level of Runx2 are somehow blocked at an early stage of differentiation that is not compatible with a normal maturation and differentiation into osteocytes. miR-199b was known to be possibly involved in the control of osteoblast differentiation by Runx2. A signal-amplification circuit between miR-218 and Wnt/β-catenin signal was reported to promote human adipose tissue-derived stem cells osteogenic differentiation [39]. In addition, miR-218/Wnt signaling circuit amplifies both the osteoblast phenotype and osteomimicry-related tumor activity [40]. MicroRNA hsa-miR-135b could regulate the mineralization in osteogenic differentiation of human unrestricted somatic stem cells [41]. Upregulation of miR-135b was known to involve in the impaired osteogenic differentiation of mesenchymal stem cells derived from multiple myeloma patients [42]. Down-regulation of miRNA-221 was discovered to trigger osteogenic differentiation in human unrestricted somatic stem cells and human mesenchymal stem cells [43]. Therefore, future investigation will be focus on the potential function of these exosomes-associated miRNAs we found in hBMSC differentiation, for example paracrine/autocrine or in communication between hBMSC/osteoblast or with other cell types.

MicroRNAs are intriguing regulatory molecules that are networked with cell signaling pathways and intricate transcriptional programs through ingenuous circuits with remarkably simple logic [36]. Prediction and identification of the miRNA-targeting genes offers an experimental basis for further research on miRNA regulatory mechanisms. Bioinformatic methods based on sequence similarities between targets and miRNAs were used to predict the potential target genes. In the present study, we utilized DIANA-mirPath to demonstrate that RNA degradation, mRNA surveillance pathway, Wnt signaling pathway, RNA transport were the most prominent pathways enriched in quantiles with differential exosomal miRNA patterns related to osteogenic differentiation. Based on the previous references in these pathways, mRNA destabilization with consequent degradation are the mechanisms by which miRNAs are believed to suppress the expression of their targets [44]. It was previously reported that degradation of ATPase inhibitory factor 1 (IF1) controls energy metabolism during osteogenic differentiation of human mesenchymal stem cells [45]. BMPs, particularly BMP2, 6 and 9, are major osteogenic growth factors that induce osteogenic differentiation in MSCs [46]. Interestingly, Wnt5a, a classical noncanonical Wnt, was recently reported as a critical component of BMP2-mediated osteogenic differentiation [34]. Others have also shown that BMPs can downregulate Wnt signaling in osteogenic differentiation via sclerostin and Dkk-1. Knocking out BMP receptor type 1 in osteoblasts led to downregulation of sclerostin and Dkk-1 and an increased bone mass phenotype in mice [47]. As an explanation for the Wnt-antagonizing effects of BMP, it was suggested that Smad1 may form a complex with Dvl, thereby sequestering Dvl from the canonical Wnt pathway [48]. However, these seemingly conflicting findings on the crosstalk between BMPs and Wnts remain unresolved. In addition, IKK–NF-κB signaling in differentiated osteoblasts has an antianabolic effect on bone formation. Time- and stage-specific inhibition of IKK-NF-κB in differentiated osteoblasts significantly enhanced bone matrix formation and mineral density during postnatal bone growth [49], [50]. NF-κB further inhibits osteogenic differentiation of mesenchymal stem cells by promoting β-catenin degradation [51]. As such, these results might indicate that exosomal miRNAs exert a moderating regulatory function in osteogenic differentiation through networking with cell signaling pathways.

Exosomes are either released from the cell when multivesicular bodies fuse with the plasma membrane or they are released directly from the plasma membrane [52]. It would be helpful to know the origin of the exosomes to evaluate the importance and function of the altered miRNAs in osteogenic differentiation of human BSMCs. However, specific markers of cellular origin are not yet available. Known exosomal surface markers, such as CD63, CD81, and CD9, are primarily used for evaluating the exosomal content of the preparation. The presence of CD63 was detected in all samples in the current study, and previous investigations demonstrated the purity of exosomes isolated by using the presence of CD63 by means of FACS. It would be important to study the origin of exosomes during osteogenic differentiation of human BSMCs in the future. It is actively researched on the role that exosomes may play in cell-to-cell signaling, hypothesizing that because exosomes can merge with and release their contents into cells that are distant from their cell of origin, they may influence processes in the recipient cell. For example, RNA that is shuttled from one cell to another, known as “exosomal shuttle RNA,” could potentially affect protein production in the recipient cell [12], [53]. By transferring molecules from one cell to another, exosomes from certain cells of the immune system, such as dendritic cells and B cells, may play a functional role in mediating adaptive immune responses to pathogens and tumors [54]. Conversely, exosome production and content may be influenced by molecular signals received by the cell of origin. As evidence for this hypothesis, tumor cells exposed to hypoxia secrete exosomes with enhanced angiogenic and metastatic potential, suggesting that tumor cells adapt to a hypoxic microenvironment by secreting exosomes to stimulate angiogenesis or facilitate metastasis to more favorable environment [55]. On the other hand, myc-immortalization of mesenchymal stem cell (MSC) did not alter the cardioprotective potency of its secreted exosomes [56]. Currently, there are no proven mechanisms by which microvesicles trigger intercellular communication. Possible mechanisms by which microvesicles trigger intercellular communication are paracrine, fusion and phagocytosis [57].

To conclude, our study reveals more details about the allocation of human BMSCs into osteogenic lineage through modulatory effect of miRNAs on targets and pathways required for creating a tissue-specific phenotype and may aid in future clinical interventions.

## Supporting Information

S1 Figure Protein expression levels of osteoblastic target genes, such as ALP, BMP-2, and PDGF-A, from human BMSCs at 0, 0.5, 1, 1.5, 2, 2.5, 3, 4, 5, 7 days. GAPDH tested was as a loading control. Data shown are representative of at least three independent experiments.
(TIF)

S2 Figure Flow cytometric analysis of BSMC exosomes displayed expression of CD63 and MHCII surface markers, whereas MHC class I, CD54, and CD86 were not detected. Results are shown as the MFI for the detected molecule divided by the MFI for the isotype control. Rows refer to time course comparison: BMSC culture at 0, 0.5, 1, 1.5, 2, 2.5, 3, 4, 5, 7 days. Data shown are representative of at least three independent experiments and are shown as mean ± SEM.
(TIF)

S1 Table The relative abundance of each miRNA (% from total) within the exosomes at the different differentiation time points.
(DOCX)
