# Supplementary figures and images for: Altered MicroRNA Expression Profile in Exosomes during Osteogenic Differentiation of Human Bone Marrow-Derived Mesenchymal Stem Cells
Source: PLoS One. 2014 Dec 11;9(12):e114627. doi: 10.1371/journal.pone.0114627 (PMC4263734; doi:10.1371/journal.pone.0114627)

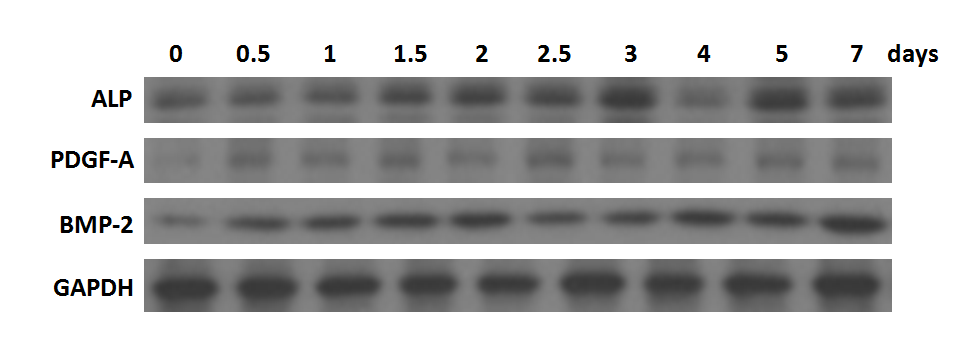

Supplement: S1 Figure — Protein expression levels of osteoblastic target genes, such as ALP, BMP-2, and PDGF-A, from human BMSCs at 0, 0.5, 1, 1.5, 2, 2.5, 3, 4, 5, 7 days. GAPDH tested was as a loading control. Data shown are representative of at least three independent experiments. (TIF) [file pone.0114627.s001.tif]

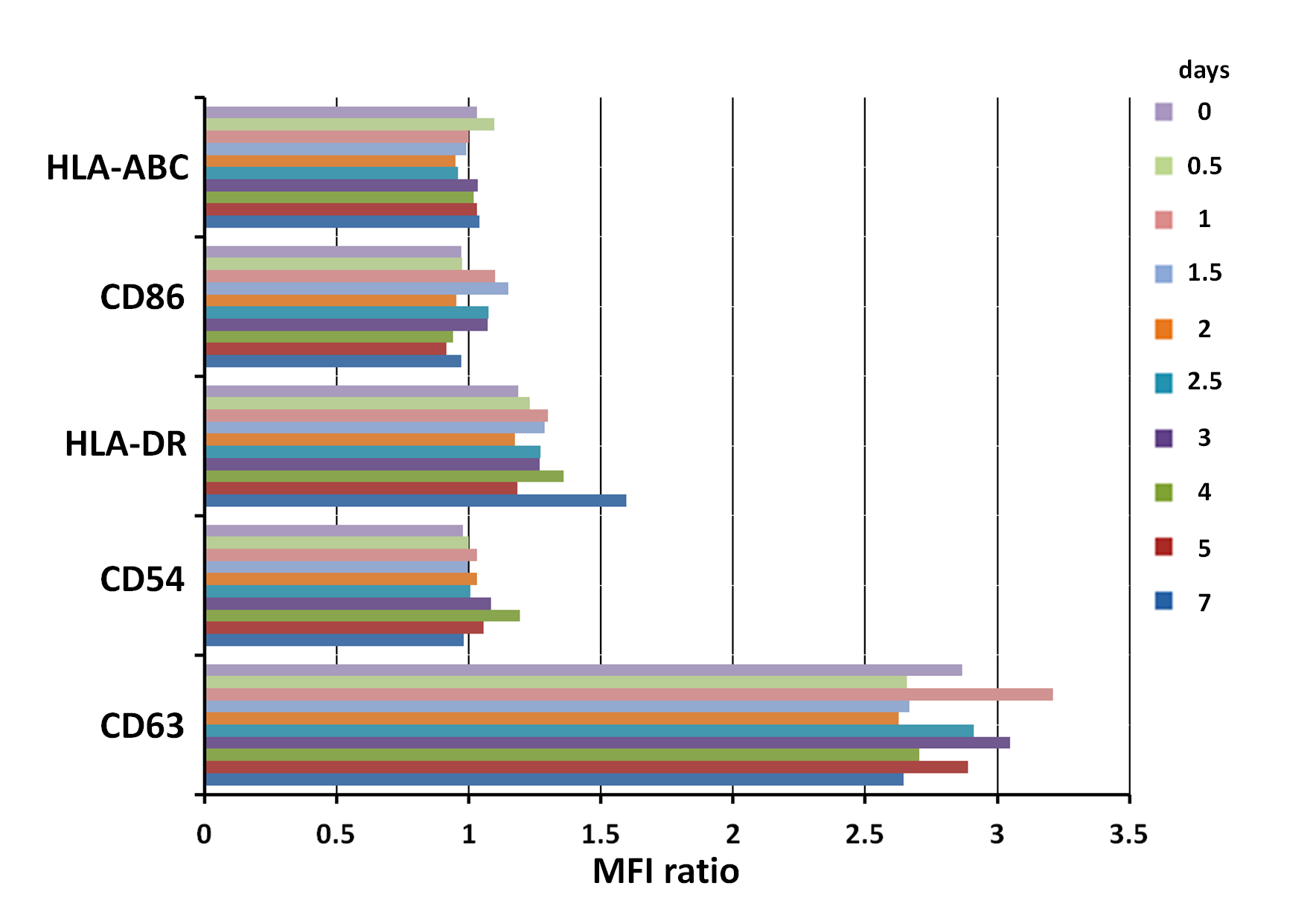

Supplement: S2 Figure — Flow cytometric analysis of BSMC exosomes displayed expression of CD63 and MHCII surface markers, whereas MHC class I, CD54, and CD86 were not detected. Results are shown as the MFI for the detected molecule divided by the MFI for the isotype control. Rows refer to time course comparison: BMSC culture at 0, 0.5, 1, 1.5, 2, 2.5, 3, 4, 5, 7 days. Data shown are representative of at least three independent experiments and are shown as mean ± SEM. (TIF) [file pone.0114627.s002.tif]
