# Supplementary material for: Altered MicroRNA Expression Profile in Exosomes during Osteogenic Differentiation of Human Bone Marrow-Derived Mesenchymal Stem Cells
Source: PLoS One. 2014 Dec 11;9(12):e114627. doi: 10.1371/journal.pone.0114627 (PMC4263734; doi:10.1371/journal.pone.0114627)
Supplement: S1 Table — The relative abundance of each miRNA (% from total) within the exosomes at the different differentiation time points. (DOCX) [file pone.0114627.s003.docx]

Supplemental table 1. The relative abundance of each miRNA (% from total) within the exosomes at the different differentiation time points.

| miRNAs | 0 | 0.5 day | 1 day | 1.5 days | 2 days | 2.5 days | 3 days | 3.5 days | 4 days | 4.5 days | 7 days |
| --- | --- | --- | --- | --- | --- | --- | --- | --- | --- | --- | --- |
| hsa-miR-154 | 0.0055 | 0.0118 | 0.0037 | 0.0197 | 0.0094 | 0.0082 | 0.0097 | 0.0052 | 0.0090 | 0.0271 | na |
| hsa-miR-203 | na | 0.0181 | 0.0141 | 0.0157 | 0.0094 | 0.0092 | 0.0160 | 0.0075 | 0.0049 | 0.0096 | na |
| hsa-miR-590-5p | 0.0108 | 0.0091 | 0.0429 | 0.0118 | 0.0094 | 0.0076 | 0.0061 | 0.0111 | 0.0137 | 0.0363 | na |
| hsa-miR-485-3p | 0.0073 | 0.0167 | 0.0129 | 0.0092 | 0.0464 | 0.0112 | 0.0174 | 0.0313 | 0.0108 | 0.0078 | 0.0022 |
| hsa-miR-518b | 0.0026 | 0.0060 | 0.0074 | 0.0095 | 0.0094 | 0.0381 | 0.0132 | 0.0393 | 0.0087 | 0.0115 | na |
| hsa-miR-511 | 0.0261 | 0.0085 | na | 0.0083 | 0.0686 | 0.0139 | na | 0.0069 | 0.0208 | 0.0040 | 0.0023 |
| hsa-miR-138 | 0.0062 | 0.0181 | 0.0080 | 0.0151 | 0.0263 | 0.0074 | 0.0031 | 0.0050 | na | na | 0.0028 |
| hsa-miR-371-3p | 0.0154 | 0.0097 | 0.0211 | 0.0092 | 0.0111 | 0.0097 | 0.0182 | 0.0262 | 0.0113 | 0.0088 | 0.0029 |
| hsa-miR-570 | 0.0089 | 0.0072 | 0.0075 | 0.0092 | 0.0094 | 0.0125 | 0.0028 | 0.0107 | 0.0062 | 0.0071 | 0.0031 |
| hsa-miR-135b | 0.0094 | 0.0223 | 0.0079 | 0.0210 | 0.0094 | 0.0241 | 0.0112 | na | 0.0096 | 0.0096 | 0.0032 |
| hsa-miR-520f | 0.0035 | 0.0056 | 0.0067 | 0.0079 | 0.0100 | 0.0112 | 0.0101 | 0.0083 | 0.0115 | 0.0096 | 0.0033 |
| hsa-miR-133b | 0.0086 | 0.0157 | 0.0161 | 0.0146 | 0.0094 | na | 0.0162 | 0.0328 | 0.0045 | 0.0079 | 0.0034 |
| hsa-miR-873 | 0.0141 | 0.0473 | 0.0129 | 0.0089 | 0.0094 | na | 0.0234 | 0.0055 | 0.0155 | 0.0093 | 0.0035 |
| hsa-miR-139-5p | 0.0136 | 0.0097 | 0.0101 | 0.0098 | 0.0094 | 0.0073 | na | 0.0057 | 0.0269 | na | 0.0035 |
| hsa-miR-654-3p | 0.0049 | 0.0091 | 0.0047 | 0.0092 | 0.0094 | 0.0152 | 0.0103 | 0.0078 | 0.0066 | 0.0273 | 0.0035 |
| hsa-miR-339-5p | 0.0285 | 0.0120 | 0.0455 | 0.0092 | 0.0094 | 0.0096 | 0.0170 | 0.0034 | 0.0147 | 0.0070 | 0.0035 |
| hsa-miR-320c | 0.0140 | 0.0107 | 0.0054 | 0.0092 | 0.0094 | 0.0090 | 0.0062 | 0.0067 | 0.0177 | 0.0037 | 0.0035 |
| hsa-miR-188-3p | 0.0148 | 0.0028 | 0.0079 | 0.0092 | 0.0428 | 0.0220 | 0.0076 | 0.0024 | 0.0064 | 0.0096 | 0.0035 |
| hsa-miR-302a | 0.0481 | 0.0110 | 0.0160 | 0.0092 | 0.0094 | 0.0077 | 0.0085 | 0.0160 | 0.0088 | 0.0096 | 0.0035 |
| hsa-miR-98 | 0.0393 | 0.0238 | 0.0165 | 0.0094 | 0.0094 | 0.0119 | 0.0024 | 0.0097 | 0.0032 | 0.0128 | 0.0037 |
| hsa-miR-509-3-5p | 0.0078 | 0.0191 | na | 0.0092 | 0.0094 | 0.0074 | 0.0100 | 0.0021 | 0.0178 | 0.0061 | 0.0041 |
| has-let-7a | 0.0082 | 0.0131 | 0.0172 | 0.0098 | 0.0094 | 0.0149 | 0.0099 | 0.0084 | 0.0244 | 0.0107 | 0.0042 |
| hsa-miR-503 | 0.0373 | 0.0078 | 0.0065 | 0.0247 | 0.0103 | 0.0074 | 0.0121 | 0.0026 | 0.0068 | 0.0039 | 0.0042 |
| hsa-miR-501-3p | 0.0078 | 0.0096 | 0.0125 | 0.0092 | 0.0094 | 0.0099 | 0.0140 | 0.0140 | na | 0.0035 | 0.0044 |
| hsa-miR-296-3p | 0.0087 | 0.0085 | 0.0034 | 0.0194 | 0.0094 | 0.0154 | na | 0.0075 | 0.0145 | 0.0183 | 0.0045 |
| hsa-miR-508 | 0.0194 | 0.0061 | 0.0169 | 0.0159 | na | 0.0237 | 0.0108 | 0.0094 | 0.0176 | 0.0105 | 0.0045 |
| ath-miR159a | 0.0088 | 0.0066 | 0.0047 | 0.0046 | 0.0126 | 0.0057 | 0.0179 | 0.0096 | 0.0467 | 0.0096 | 0.0046 |
| hsa-miR-373 | 0.0088 | 0.0338 | 0.0063 | 0.0092 | 0.0094 | 0.0097 | 0.0270 | 0.0124 | 0.0083 | 0.0096 | 0.0046 |
| hsa-miR-199b | 0.0093 | 0.0101 | 0.0129 | 0.0038 | 0.0125 | 0.0107 | 0.0125 | 0.0273 | 0.0069 | 0.0160 | 0.0048 |
| hsa-miR-221 | 0.0109 | 0.0081 | 0.0018 | 0.0082 | 0.0094 | na | 0.0118 | 0.0071 | 0.0118 | 0.0099 | 0.0048 |
| hsa-miR-561 | 0.0088 | na | na | 0.0105 | 0.0094 | 0.0124 | 0.0381 | 0.0075 | 0.0043 | 0.0156 | 0.0052 |
| hsa-miR-422a | 0.0111 | 0.0073 | 0.0046 | 0.0507 | 0.0094 | 0.0064 | 0.0076 | 0.0099 | 0.0063 | 0.0074 | 0.0063 |
| hsa-miR-499-3p | na | 0.0101 | 0.0639 | 0.0041 | 0.0094 | 0.0326 | 0.0083 | 0.0034 | 0.0083 | 0.0118 | 0.0065 |
| hsa-miR-510 | 0.0088 | 0.0430 | 0.0154 | 0.0092 | 0.0094 | 0.0085 | na | 0.0044 | 0.0047 | 0.0050 | 0.0080 |
| hsa-miR-556-3p | 0.0088 | 0.0141 | 0.0211 | 0.0431 | na | 0.0126 | 0.0025 | 0.0143 | 0.0126 | 0.0056 | 0.0080 |
| hsa-miR-129 | 0.0088 | 0.0086 | 0.0043 | 0.0063 | 0.0094 | 0.0162 | 0.0087 | 0.0070 | 0.0053 | 0.0062 | 0.0080 |
| hsa-miR-625 | 0.0018 | 0.0111 | 0.0090 | 0.0064 | 0.0094 | 0.0087 | 0.0035 | na | 0.0088 | 0.0079 | 0.0080 |
| hsa-miR-548c | 0.0088 | 0.0079 | 0.0066 | 0.0092 | 0.0359 | 0.0169 | 0.0122 | 0.0073 | 0.0123 | 0.0087 | 0.0080 |
| hsa-miR-148a | 0.0088 | 0.0118 | 0.1098 | 0.0231 | 0.0094 | 0.0062 | 0.0088 | 0.0096 | 0.0087 | 0.0107 | 0.0080 |
| hsa-miR-95 | 0.0026 | 0.0241 | 0.0152 | 0.0060 | 0.0094 | 0.0111 | na | 0.0040 | 0.0134 | 0.0487 | 0.0080 |
| hsa-miR-674 | 0.0045 | 0.0126 | 0.0028 | 0.0230 | 0.0094 | 0.0188 | 0.0034 | 0.0163 | 0.0138 | 0.0094 | 0.0083 |
| hsa-miR-181a | 0.0161 | 0.0082 | 0.0029 | 0.0103 | 0.0097 | 0.0052 | 0.0116 | 0.0177 | 0.0025 | 0.0245 | 0.0089 |
| hsa-miR-339-3p | 0.0171 | 0.0156 | 0.0122 | 0.0092 | 0.0446 | 0.0050 | 0.0103 | 0.0045 | 0.0063 | 0.0073 | 0.0147 |
| hsa-miR-483-5p | 0.0210 | 0.0109 | 0.0459 | 0.0092 | 0.0094 | 0.0088 | 0.0111 | 0.0596 | 0.0079 | 0.0352 | 0.0147 |
| hsa-miR-579 | 0.0065 | 0.0119 | 0.0214 | 0.0085 | 0.0094 | 0.0424 | 0.0271 | na | 0.0120 | 0.0171 | 0.0148 |
| hsa-miR-876-3p | 0.0191 | 0.0079 | 0.0115 | 0.0106 | 0.0094 | 0.0092 | 0.0049 | 0.0048 | na | 0.0172 | 0.0149 |
| hsa-miR-362-3p | 0.0190 | 0.0119 | 0.0133 | 0.0092 | 0.0094 | 0.0160 | 0.0194 | na | 0.0303 | 0.0071 | 0.0165 |
| hsa-miR-500 | 0.0127 | 0.0177 | 0.0013 | 0.0092 | 0.0065 | 0.0089 | 0.0102 | 0.0164 | 0.0083 | 0.0106 | 0.0166 |
| hsa-miR-598 | 0.0172 | 0.0133 | 0.0040 | na | 0.0140 | 0.0125 | 0.0113 | 0.0075 | 0.0089 | 0.0193 | 0.0166 |
| hsa-miR-545 | 0.0154 | 0.0054 | 0.0073 | 0.0155 | 0.0094 | 0.0031 | 0.0130 | 0.0081 | na | 0.0193 | 0.0167 |
| hsa-miR-453 | 0.0041 | 0.0090 | 0.0130 | 0.0121 | 0.0094 | 0.0035 | 0.0346 | 0.0116 | 0.0097 | 0.0096 | 0.0176 |
| hsa-miR-367 | 0.0072 | 0.0114 | 0.0097 | 0.0092 | na | 0.0087 | 0.0590 | 0.0066 | 0.0343 | 0.0096 | 0.0177 |
| hsa-miR-193a-3p | 0.0122 | na | na | 0.0092 | 0.0172 | 0.0066 | 0.0244 | 0.0273 | 0.0075 | 0.0144 | 0.0178 |
| hsa-miR-548c-5p | 0.0122 | 0.0031 | 0.0024 | 0.0092 | 0.0172 | 0.0066 | 0.0244 | 0.0273 | 0.0075 | 0.0144 | 0.0178 |
| hsa-miR-326 | 0.0099 | 0.0109 | 0.0025 | 0.0092 | 0.0118 | 0.0085 | 0.0211 | 0.0115 | 0.0105 | 0.0092 | 0.0178 |
| hsa-miR-218 | 0.0114 | 0.0261 | 0.0030 | 0.0216 | 0.0094 | 0.0071 | 0.0678 | 0.0161 | 0.0118 | 0.0096 | 0.0200 |
| hsa-miR-219 | 0.0081 | 0.0117 | 0.0175 | 0.0099 | 0.0094 | 0.0073 | 0.0159 | 0.0075 | 0.0178 | 0.0096 | 0.0202 |
| hsa-miR-885-5p | 0.0205 | 0.0097 | 0.0093 | 0.0077 | 0.0094 | 0.0113 | 0.0093 | 0.0187 | 0.0096 | 0.0096 | 0.0203 |
| hsa-miR-377 | 0.0229 | 0.0110 | 0.0253 | 0.0043 | 0.0089 | 0.0028 | 0.0148 | 0.0228 | 0.0051 | 0.0096 | 0.0203 |
| hsa-miR-216a | 0.0114 | 0.0077 | 0.0505 | 0.0329 | 0.0436 | 0.0555 | 0.0085 | 0.0472 | 0.0369 | 0.0030 | 0.0226 |
| hsa-miR-512-3p | 0.0092 | 0.0068 | 0.0140 | 0.0503 | 0.0094 | 0.0162 | 0.0085 | 0.0099 | 0.0205 | 0.0029 | 0.0226 |
| hsa-miR-493 | 0.0374 | 0.0037 | 0.0062 | 0.0092 | 0.0094 | 0.0067 | 0.0093 | 0.0160 | 0.0187 | 0.0173 | 0.0232 |
| hsa-miR-324-3p | 0.0216 | 0.0099 | 0.0142 | 0.0092 | 0.0077 | 0.0111 | 0.0085 | 0.0023 | 0.0037 | 0.0195 | 0.0235 |
| hsa-miR-302b | 0.0095 | 0.0120 | 0.0107 | 0.0081 | 0.0133 | 0.0178 | 0.0066 | 0.0197 | 0.0093 | 0.0173 | 0.0236 |
| hsa-miR-149 | 0.0132 | 0.0119 | 0.0062 | 0.0058 | 0.0094 | 0.0142 | 0.0170 | 0.0125 | 0.0105 | 0.0195 | 0.0236 |
| hsa-miR-517b | 0.0305 | 0.0040 | 0.0307 | 0.0112 | 0.0094 | 0.0450 | 0.0079 | 0.0083 | 0.0052 | 0.0287 | 0.0237 |
| hsa-miR-219-1-3p | 0.0068 | 0.0102 | 0.0105 | 0.0167 | 0.0050 | 0.0713 | 0.0057 | 0.0185 | 0.0130 | na | 0.0237 |
| hsa-miR-383 | na | 0.0371 | 0.0060 | 0.0092 | 0.0094 | 0.0081 | 0.0118 | 0.0167 | 0.0189 | 0.0114 | 0.0237 |
| hsa-miR-33b | 0.0029 | 0.0189 | 0.0151 | 0.0092 | 0.0151 | 0.0090 | 0.0028 | 0.0074 | 0.0083 | 0.0163 | 0.0238 |
| hsa-miR-519e | 0.0036 | 0.0107 | 0.0087 | 0.0092 | 0.0094 | 0.0034 | 0.0053 | 0.0089 | 0.0336 | 0.0014 | 0.0238 |
| hsa-miR-518a-3p | 0.0477 | 0.0139 | 0.0073 | na | 0.0094 | 0.0083 | 0.0211 | 0.0079 | 0.0438 | 0.0106 | 0.0250 |
| hsa-miR-299-5p | 0.0119 | 0.0177 | 0.0079 | 0.0217 | 0.0328 | 0.0393 | 0.0166 | 0.0600 | 0.0107 | 0.0182 | 0.0250 |
| hsa-miR-208 | 0.0084 | na | 0.0137 | 0.0062 | 0.0094 | 0.0088 | 0.0071 | 0.0326 | 0.0086 | 0.0101 | 0.0252 |
| hsa-miR-105 | 0.0181 | 0.0094 | 0.0151 | 0.0427 | 0.0120 | na | 0.0076 | 0.0422 | 0.0472 | 0.0096 | 0.0265 |
| hsa-miR-509-5p | 0.0127 | 0.0233 | 0.0120 | 0.0070 | 0.0094 | 0.0101 | 0.0085 | 0.0094 | 0.0347 | 0.0096 | 0.0266 |
| hsa-miR-34a | 0.0115 | 0.0105 | 0.0055 | 0.0092 | 0.0094 | 0.0029 | 0.0115 | 0.0099 | 0.0075 | 0.0070 | 0.0267 |
| hsa-miR-450b-5p | 0.0102 | 0.0106 | 0.0108 | 0.0092 | 0.0094 | 0.0074 | 0.0170 | 0.0092 | 0.0120 | 0.0110 | 0.0281 |
| hsa-miR-380-3p | 0.0085 | 0.0176 | 0.0269 | 0.0108 | 0.0136 | 0.0091 | 0.0085 | 0.0071 | 0.0060 | 0.0096 | 0.0283 |
| hsa-miR-155 | 0.0106 | 0.0083 | na | na | 0.0094 | 0.0084 | 0.0085 | na | 0.0089 | 0.0096 | 0.0286 |
